# Green Preparation of High Yield Fluorescent Graphene Quantum Dots from Coal-Tar-Pitch by Mild Oxidation

**DOI:** 10.3390/nano8100844

**Published:** 2018-10-17

**Authors:** Quanrun Liu, Jingjie Zhang, He He, Guangxu Huang, Baolin Xing, Jianbo Jia, Chuanxiang Zhang

**Affiliations:** 1 College of Chemistry and Chemical Engineering, Henan Polytechnic University, Jiaozuo 454003, China; zhangjingjie1221@163.com (J.Z.); hpuhehe@163.com (H.H.); guangxu1369@163.com (G.H.); baolinxing@hpu.edu.cn (B.X.); jiajianbo@hpu.edu.cn (J.J.); 2 Collaborative Innovation Center of Coal Work Safety of Henan Province, Jiaozuo 454003, China; 3 Henan Key Laboratory of Coal Green Conversion, Jiaozuo 454003, China

**Keywords:** graphene quantum dots, coal tar pitch, luminescence, green

## Abstract

Coal tar pitch (CTP), a by-product of coking industry, has a unique molecule structure comprising an aromatic nucleus and several side chains bonding on this graphene-like nucleus, which is very similar to the structure of graphene quantum dots (GQDs). Based on this perception, we develop a facile approach to convert CTP to GQDs only by oxidation with hydrogen peroxide under mild conditions. One to three graphene layers, monodisperse GQDs with a narrow size distribution of 1.7 ± 0.4 nm, are obtained at high yield (more than 80 wt. %) from CTP. The as-produced GQDs are highly soluble and strongly fluorescent in aqueous solution. This simple strategy provides a feasible route towards the commercial synthesis of GQDs for its cheap material source, green reagent, mild condition, and high yield.

## 1. Introduction

In recent years, graphene has attracted more and more attention due to its unique properties [1], such as excellent mechanical properties [2], thermal properties [3] and electronic properties [4]; it has a wide range of applications on optoelectronics, energy storage, biomedical, catalysis, sensors and among many others [5,6,7,8]. As the latest member of the graphene family, graphene quantum dots (GQDs), which can be a promising alternative to traditional semiconductor quantum dots, show promising applications and potential developments in terms of bioimaging, electrochemical biosensors, photovoltaic devices and in the biomedical field, among many others [9,10,11,12,13,14,15,16]. GQDs are generally derived from glucose, carbon fibers, graphite, graphene oxides, and synthesized or fabricated by methods like electrochemical oxidation, lithographic patterning, hydrothermal, microwave, acidic oxidation and supercritical fluid treatment [15,17,18,19,20,21,22,23,24,25,26,27,28]. The high price of the carbon source (graphite, carbon fibers, and carbon nanotubes), long processing time (24–48 h), post-purification procedure (3–5 days) and the harsh reaction conditions (nitrating mixture or oxidizing supercritical water) for the large-scale production of GQDs are uneconomic and unreasonable, so it is imperative and desirable to prepare GQDs in a more facile, milder and more environmentally-friendly method [18,19,21,23,24,29,30]. For example, Ye et al. have reported a wet-chemistry route to fabricate graphene quantum dots by heat treatment of coal in a nitrating mixture at 120 °C for 24 h, followed by a series of neutralization, filtration and dialysis (for ~5 days) procedures [21]. Recently, Sasikala et al. extracted graphene quantum dots from coal by supercritical water treatment at 400 °C and 25 MPa for 2 h [23]. However, the former approach needed long processing time, harsh reaction conditions and long post treatment purification procedures, which is time-consuming and environmentally unfriendly; the latter required supercritical condition to treat samples, but its high temperature and high pressure operating conditions undoubtedly place heavy demands on the equipment.

Coal tar pitch (CTP) is a residue of coal tar distilling and extracting (such as light oil, phenol oil, naphthalene oil, wash oil and anthracene oil), accounting for about 50% to 60% in the total coal tar. The basic structural of CTP molecular is making up a polyaromatic hydrocarbons nucleus and several alkyl side chains or heteroatoms functional groups bonding on the aromatic nucleus [31]. Furthermore, polyaromatic hydrocarbons (PAHs) are well-defined “pieces of graphite” [32]; therefore, the molecular structure of CTP is very similar to the structure of graphene quantum dots (GQDs) [33]. So, by comparing with other starting materials, CTP should be easily converted to graphene quantum dots. Most recently, Meng et al. fabricated carbon dots (CDs) by the reaction of coal pitch with solution of formic acid and H_2_O_2_, but they found that the formation of CDs highly relies on the blending ration of formic acid and H_2_O_2_ [34].

In this work, we reported a facile one-step green route for the fabrication of GQDs, and demonstrated that by simply heating the mixture of CTP and hydrogen peroxide to reflux for 120 min, we can obtain high-yield GQDs with narrow size distribution. Our methodology does not require harsh reagents/production environments, elaborate synthesis conditions, long reaction time or post-treatment purification procedures, and can be extended in an environmentally-friendly way to mass production. Under the effect of H_2_O_2_ oxidation, CTP with cheap price and stacked graphitic layers were selectively converted to well-dispersed CQDs.

## 2. Materials and Methods

### 2.1. Materials

CTP was directly purchased from Henan Zhonghong Coal Chemical Co., Ltd. (Pingdingshan, China), and hydrogen peroxide aqueous solution (30 wt. %) was of analytical-reagent grade and purchased from Sinopharm Chemical Reagent Co., Ltd. (Shanghai, China). Deionized water with a resistivity of 18.1 MΩ cm was used for all experiments. Copper-carbon grids were purchased from Beijing Zhongjingkeyi Technology Co., Ltd. (Beijing, China). Polyethersulfone filter membranes (0.22 μm) were purchased from Jinteng Experimental Equipment Co., Ltd. (Tianjin, China).

### 2.2. Preparation of GQDs

In a typical procedure, 200 milligrams of CTP were suspended in hydrogen peroxide (200 mL), and followed by cup sonication for 2 h. The obtained reaction mixture was then stirred and heated at 100 °C in reflux for 2 h in a round-buttom flask. The color of solution changed from black brown to deep yellow solution after 2 h, implying the formation of GQDs. The solution was cooled to room temperature and poured into a beaker. The solution was then filtered through a 0.22 μm filter membrane to remove the insoluble larger fragments. The filtrate was concentrated using vacuum freeze drying to obtain solid GQDs.

### 2.3. Characterization of GQDs

SEM was performed on a Zeiss Merlin Compact high-resolution field emission (Carl Zeiss, Jena, Germany), 5 nm Au was sputtered on the CTP surface before imaging. The TEM and HRTEM images were obtained on a Tecnai G2 20 200 kV TEM (FEI, Hillsboro, OR, USA) and samples were prepared by depositing a drop of GQDs suspensions onto copper-carbon grids. The AFM image was obtained on a Dimension Edge AFM (Bruker, Karlsruhe, Germany). The GQDs aqueous solutions were spin coated (3000 rpm) onto a freshly cleaved mica substrate and dried at room temperature before imaging. XPS analyses were carried out on a Thermo escalab 250Xi X-ray photoelectron spectrometer (Thermo Fisher Scientific, Waltham, MA, USA) with a chamber pressure of 3 × 10^−9^ mBar and a monochromatic Al Kα source (1486.6 eV) as the X-ray source. The source power was set at 150 W, and pass energies of 100.0 eV for survey scans and 20.0 eV for high-resolution scans were used. Raman spectrum of CTP was recorded using a Renishaw in Via Microscopic Confocal Raman Spectrometer (Renishaw plc, Gloucestershire, UK) under Ar ion laser with an excitation wavelength of 532 nm at room temperature. The spectrum was calibrated in frequency using a piece of silicon prior to measurement. FTIR spectra were recorded on a Bruker Tensor 27 vacuum FTIR spectrometer (Bruker, Berlin, Germany) using KBr pellets. Ultraviolet-visible spectra were recorded on a Shimadzu UV-2450 ultraviolet-visible spectrophotometer (Shimadzu, Kyoto, Japan). Photoluminescence spectra were recorded using a luminescence spectrometer (Nanolog FL3-2iHR, Horiba Jobin Yvon, Paris, France) with xenon lamp as the source of excitation.

### 2.4. Product GQDs Yields Calculation

The yields of GQDs could be calculated using the following formula: Yield (%) = M_GQDs_/M_CTP_ × 100%, where M_GQDs_ is the mass of the collected solid GQDs and M_CTP_ is the mass of the CTP used to prepare GQDs.

### 2.5. Relative QY Measurements

The quantum yields (QY) of GQDs were calculated according to the following equation [35]: *Φ_i_* = *Φ_s_F_i_f_s_ƞ_i_*^2^/*F_s_f_i_ƞ_s_*^2^, where *Φ_i_* and *Φ_s_* are the photoluminescence QY of the sample and that of the standard, respectively. *F_i_* and *F_s_* are the integrated intensities (areas) of sample and standard spectra, respectively (in units of photons); *f_x_* is the absorption factor, the fraction of the light impinging on the sample that is absorbed (*f_x_* = 1 − 10^−*A*^_x_, where *A* = absorbance); the refractive indices of the sample and reference solution are *ƞ_i_* and *ƞ_s_*, respectively.

### 2.6. Energy Gap Calculation

*E* = *hc*/*λ*, where *h* is the Planck constant; *c* is the speed of light; *λ* is the wavelength of absorption or emission.

## 3. Results and Discussion

The macro image and simplified molecular structure of CTP are given in Figure S1. The surface elements and functional groups of the CTP were characterized by X-ray photoelectron spectroscopy (XPS) and summed up in Supplementary Figure S2a,b and Tables S1 and S2. The XPS shows that CTP has high carbon content (93.54%) and trace inorganic minerals content. The C1s high-resolution XPS reveals that there are high Csp^3^ content due to their abundant side chains and other amorphous carbon linking on the edges of CTP molecules, which are easier to oxidize than pure sp^2^-carbon. The solid-state Fourier transform infrared spectroscopy (ssFTIR) spectrum (Figure S2c) shows the presence of aromatic H–Csp^2^ (750 and 3040 cm^−1^), C–O stretch (1032 cm^−1^), C=C stretch (1593 cm^−1^), aliphatic H–Csp^3^ (2918 cm^−1^) and O–H (3426 cm^−1^) vibrations, which is consistent with XPS results. The Raman spectrum (Figure S2d) shows characteristic ordered D band at 1355 cm^−1^ and disordered G band at 1575 cm^−1^, while no apparent 2D and 2G peak is observed. The above results suggest that the CTP molecules contain a high proportion of disorder structure, despite the presence of graphite-like domains. The inherent disorder structure of CTP molecules makes them easier to oxidize and exfoliated than pure graphite at mild oxidative reaction conditions.

**Figure 1:**
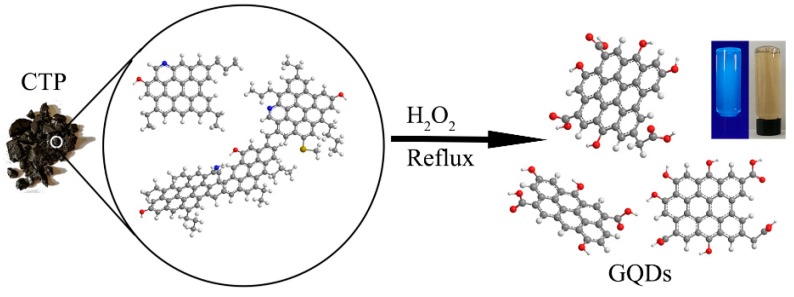
Schematic illustration of the fabrication of GQDs.

Figure 1 shows schematic illustration of the fabrication of GQDs. As a reference experiment, firstly the CTP was ultrasonicated in hydrogen peroxide (15%) for 120 min at ambient temperature. The TEM image shows that ultrasonic treatment could not exfoliate the aggregation of CTP molecules, and just led to the dispersion of the CTP as small flakes of several hundred nanometers in size (Figure S3) in the hydrogen peroxide. The obtained flakes did not show any observable photoluminescence under excitation with a UV lamp (365 nm). In contrast, the mixture of CTP and hydrogen peroxide was ultrasonicated for 120 min, and heated to reflux gently with stirring at about 100 °C for 120 min; then, the dark mixture converted to deep yellow transparent solution (see schematic illustration and Figure S4), which resulted in highly-soluble and fluorescent GQDs-1.

**Figure 2:**
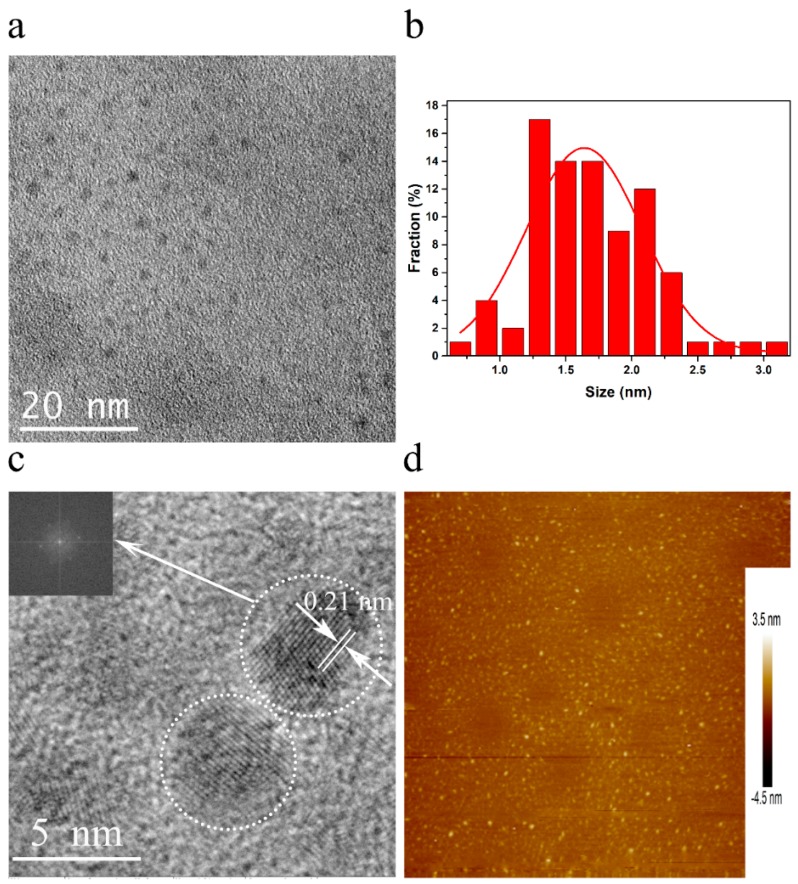
TEM and AFM images of GQDs-1. (**a**) TEM image of GQDs-1 displaying a regular size and distribution. Scale bar, 20 nm. (**b**) The size distribution of GQDs-1 in (**a**). (**c**) HRTEM image of representative GQDs-1 from (**a**); the inset is the 2D FFT pattern showing the crystalline hexagonal structure of these quantum dots corresponding to hexagonal graphene lattice fringes. Scale bar, 5 nm. (**d**) AFM image of GQDs-1 deposited on freshly cleaved mica substrates.

The microstructure of GQDs-1 was investigated by transmission electron microscopy (TEM) (Figure 2a), showing that the as-made GQDs-1 with uniformly-distributed sizes and shapes which are 1.7 ± 0.4 nm in diameter (Figure 2b). The high-resolution TEM (HRTEM) image (Figure 2c) of representative GQDs-1 particle shows hexagonal lattice in honeycomb network. The fast Fourier transform (FFT) pattern of corresponding GQDs-1 (inset in Figure 2c) reveals six spots arranged in a hexagonal pattern with a lattice parameter of 0.21 nm corresponding to the (100) plane of graphene. The atomic force microscope (AFM) image (Figure 2d) reveals the topographic morphology of GQDs-1, the average thickness of which are mostly between 1.0 and 2.0 nm (Figure S5), corresponding to one to three layers of graphene structures [24].

**Figure 3:**
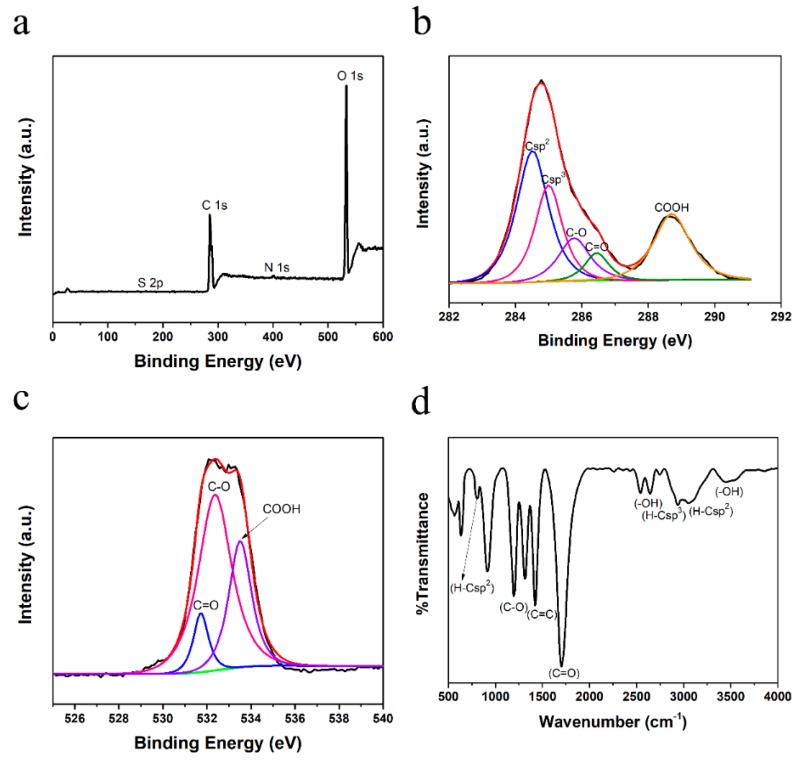
Characterization of GQDs-1. (**a**) XPS survey spectrum of GQDs-1. (**b**) High-resolution XPS C 1s spectrum of GQDs-1; a new peak corresponding to COOH appears at 288.6 eV. (**c**) The high-resolution O 1s XPS spectrum of GQDs-1. (**d**) ssFTIR spectrum of GQDs-1, obtained after evaporation of water.

The GQDs-1 exhibit high solubility and stability in water and other polar solvents such as ethanol, dimethylformamide (DMF), tetrahydrofuran (THF) and dimethyl sulfoxide (DMSO), due to the introduction of oxygen-containing functional groups. The XPS (Figure 3a) measurements were carried out to probe the chemical composition of GQDs-1, which are summed up in Table S1. The high-resolution C 1s XPS (Figure 3b and Table S2) shows the presence of Csp^2^ (284.5 eV), Csp^3^ (285.0 eV), C–O (285.8 eV), C=O (286.5 eV) and COOH (288.7 eV) peaks. This conclusion can also be obtained from the high-resolution O 1s XPS (Figure 3c) of GQDs-1. The ssFTIR spectrum (Figure 3d) is consistent with the XPS results, displaying the presence of oxygen-containing groups, including carbonyl groups (C=O), carboxyl groups (COOH), hydroxyl groups (OH) and C–O. It should be noted that the peak corresponding to the H–Csp^3^ (2940 cm^−1^) weakened evidently comparing to CTP (Figure S2d), suggesting that most side chains of the CTP were oxidized to oxygen-containing functional group, especially carboxyl groups. All of the results suggest that the CQDs derived from CTP are mainly composed of sp^2^ graphitic carbons with sp^3^ carbon defects and oxygen-rich edges such as hydroxyl, carbonyl and carboxyl groups that are abundant on the surface. Furthermore, the carboxyl and hydroxyl groups at their edge enable them to display suitability for successive functionalization with various organic, inorganic, polymeric or biological species [36]. Comparing the structures of GQDs and CTP, we speculate that the mechanism for producing GQDs from CTP in this way is as follows: the alkyl chains linking on the edges of the CTP molecules are selectively oxidized to oxygen-containing groups by H_2_O_2_, while the aromatic nucleus in the CTP molecules are preserved and formed graphite domain of the GQDs. This mechanism is different from the conventional “top-down” and “bottom-up” synthetic approaches for GQDs. The yield of GQD-1 from CTP exceeds 80 wt. % (noting that oxidation has increased the weight of the final structure). To the best of our knowledge, such high yields in fabricating GQDs have not been reported in the literature.

In another test, 0.5 g CTP and 200 mL H_2_O_2_ (30%) was treated as the similar procedure as preparation of GQDs-1 to enhance the production efficiency. After heating in reflux and constant stirring for about 60 min, the dark mixtures became transparent, which resulted in GQDs-2. The microstructure of GQDs-2 derived from the CTP was observed by TEM; the image (Figure S6a) displays their size distribution of 2.3 ± 0.7 nm (Figure S6b), a little larger and broader than GQDs-1, owing to slight aggregation for the high concentration. The HRTEM image (Figure S6c) indicates high crystallinity of the GQDs-2, with a spacing of 0.21 nm. The AFM image (Figure S6d) shows that their typical topographic heights are 1.9 ± 0.7 nm (Figure S7), suggesting that there are one to four layers of graphene structures. As for GQDs-2, they are also highly soluble and fluorescent in aqueous solution. XPS and ssFTIR spectra (Figure S8a–d and Tables S1 and S2) indicate that GQDs-2 have higher oxygen content and more oxygen-containing functional groups due to the oxidation of high concentrations of H_2_O_2_. Both GQDs-1 and GQDs-2 are rich of hydrophilic groups such as carboxyl and hydroxyl groups that are formed in the chemical oxidation step of CTP in hydrogen peroxide. The abundant hydrophilic groups greatly improve the aqueous solubility of the CQDs and support their applications in aqueous systems [37]. Compared to graphene or graphene-based nanomaterials, GQDs have better water solubility, and can combine with a variety of compounds by the intermolecular π–π interaction [15]. The functional groups of GQDs exert significant influence on their cellular penetration capability and intracellular localization, which have a potential application in the field of bioimaging [12].

**Figure 4:**
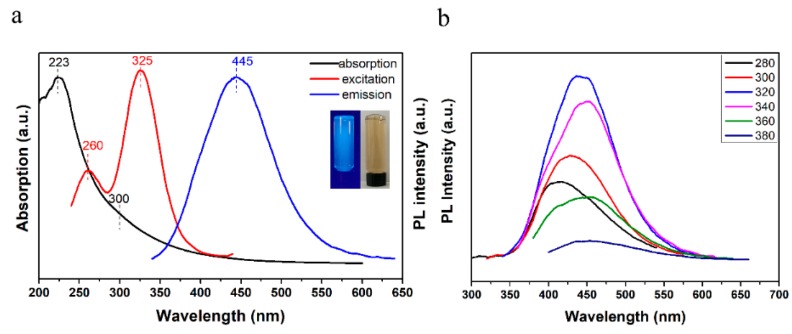
Optical characterizations of GQDs-1. (**a**) Combined UV-Vis absorption (black line), PLE spectrum with detection wavelength of 445 nm (red line) and PL spectrum excited at 325 nm (blue line) of the GQDs-1 dispersed in water. Inset of panel (**a**): the left is a photograph of the corresponding GQDs-1 aqueous solution under UV light with 365 nm excitation; the right is a photograph of the corresponding GQDs-1 aqueous solution taken under visible light. (**b**) PL spectra of the GQDs-1 solution under different excitation wavelengths.

The absorption and photoluminescence (PL) measurements of GQDs in aqueous solution were analyzed by UV-Vis and PL spectra. Figure 4a indicates that GQDs-1 in aqueous solution have two typical UV-Vis absorption peaks at 223 nm, 300 nm (black line) and display optimal excitation spectrum (red line) and the corresponding emission spectrum (blue line). The obvious absorption peak at 223 nm is ascribed to π–π* transition of C=C and another shoulder peak at 300 nm, which is assigned to the typical absorption for the n–π* transition of C=O bond [38]; this is consistent with the results of XPS and ssFTIR characterizations. The presence of these absorption peaks indicates that the GQDs-1 form a π–electron state on the substrate as graphene. In addition, there is a wide tail at 350 nm to 500 nm, which is caused by defects such as the presence of oxygen-containing functional groups. GQDs-1 possess the optimal excitation and emission wavelengths at ca. 325 nm and 445 nm, respectively, and shows a bright blue color under a UV lamp (Figure 4a). Using quinine sulfate (QY 0.54 in 0.1 M H_2_SO_4_) as the reference, the PL quantum yield of the GQDs-1 in aqueous solution was measured to be 2.37%, which is similar to those of reported luminescent carbon nanoparticles [24,26,39,40]. Like most carbon-based fluorescent materials [24,41], the excitation wavelength-dependent PL from the GQDs-1 was also observed. Figure 4b shows the photoluminescence spectra of GQDs-1 excited at different wavelengths. When the excitation wavelength is changed from 280 nm to 380 nm, the PL peak shifts to longer wavelength, and its intensity increases initially and then decreases. This excitation dependence property may result from optical selection of differently sized GQDs-1 and/or different emissive sites on GQDs-1. The photoluminescence excitation (PLE) spectrum (Figure 4a) recorded with the strongest luminescence shows two peaks: 260 nm and 325 nm. The 325-nm PLE peak corresponds to the 300-nm absorption band of GQDS-1, whereas the corresponding absorption band of the 260-nm PLE peak is hidden in the strong background absorption from the π–π* transition.

As for GQDs-2, Figure S9a shows that GQDs-2 in aqueous solution have two typical UV-Vis absorption peaks at 218 nm, 300 nm, corresponding to π–π* transition of C=C and n–π* transition of C=O, respectively. Figure S9b shows that GQDs-2 also exhibit an excitation-dependent PL behavior. The photoluminescence excitation (PLE) spectrum (Figure S10) recorded with the strongest luminescence shows two peaks: 272 nm and 328 nm.

**Figure 5:**
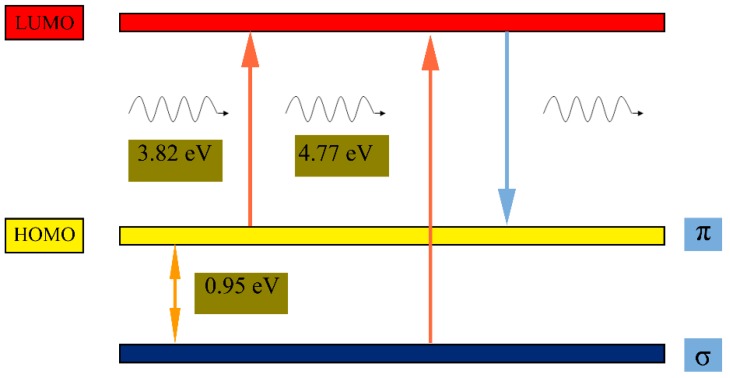
Typical electronic transitions of triple carbenes at zigzag sites of GQDs-1.

According to previous reports [24,29], the fluorescence of GQDs may originate from emissive free zigzag sites with a carbene-like triplet ground state described as σ^1^π^1^. Due to the smaller diameter (about 1.7 nm), GQDs-1 emit strong luminescence most likely because of the presence of high concentrations of free tortuous position. The carbene ground state multiplicity is related to the energy difference (*δE*) between σ and π orbitals, and Hoffmann determined that for a triplet ground state, *δE* should be below 1.5 eV [42,43]. Here, the two electronic transitions of 325 nm (3.82 eV) and 260 nm (4.77 eV) observed in the PLE spectrum can be considered as a transition from the σ and π orbitals, that the highest occupied molecular orbital (HOMO) to the lowest unoccupied molecular orbital (LUMO), as illustrated in Figure 5. The *δE* is thus determined to be 0.95 eV, which is within the required value (<1.5 eV) for triple carbenes, and which suggests that the assignment of the two transitions is reasonable. Because the two transitions are directly correlated with the observed blue PL, the blue emission is the irradiation decay of activated electrons from the LUMO to the HOMO. Thus, the observed blue PL of GQDs-1 here are related to the two transitions: the blue emission was the irradiation decay of activated electrons from the LUMO to the HOMO.

## 4. Conclusions

In this study, we have developed a simple, green and efficient strategy for the fabrication of nanometer-sized GQDs by heating the mixture of CTP and hydrogen peroxide at 100 °C for 2 h in reflux. The extraction of GQDs from CTP does not require harsh reagents/equipment, elaborate synthesis conditions, long reaction times or post-treatment purification procedures. The as-prepared GQDs with graphene-like structures possess a large numbers of oxygen-functional groups, which greatly enhance the solubility of GQDs in aqueous solutions and support their applications in aqueous systems. This simple, green process paves the way for the mass production of fluorescent GQDs.

## Supplementary Materials

The following are available online at http://www.mdpi.com/2079-4991/8/10/844/s1, Figure S1: Macroscale image and simplified illustrative molecular structure of CTP, Figure S2: Characterization of CTP. (a) XPS survey of CTP. (b) High resolution C 1s XPS spectrum of CTP displayed presence of Csp^2^, Csp^3^ and C–O modes at 284.6 eV, 285.1 eV and 285.4 eV, respectively. (c) ssFTIR spectrum of CTP showing H–Csp^2^, C–O, C=C, H–Csp^3^ and O–H vibration modes at 750/3040 cm^−1^, 1032 cm^−1^, 1593 cm^−1^, 2917 cm^−1^ and 3426 cm^−1^ as labeled, respectively. (d) Raman spectrum of CTP under excitation of 532 nm, Figure S3: TEM image of CTP after ultrasonication in hydrogen peroxide for 2 h. Inset is the optical image, Figure S4: Preparation procedure of GQDs-1, Figure S5: The height distribution of the GQDs-1, Figure S6: Characterization of GQDs-2. (a) TEM image of GQDs-2 displaying a regular size and shape distribution. Scale bar, 20 nm. (b) The size distribution histogram of GQDs-2. (c) HRTEM image of representative GQDs-2 from (a). (d) AFM image of GQDs-2, Figure S7: The height distribution of the GQDs-2, Figure S8: Characterization of GQDs-2. (a) XPS survey of GQDs-2. (b) High-resolution C 1s XPS spectrum of GQDs-2. (c) The high-resolution O 1s XPS spectrum of GQDs-2. (d) ssFTIR spectrum of GQDs-2 showing different vibration modes as labeled, Figure S9: Optical properties of the GQDs-2. (a) UV-Visible spectrum of the GQDs-2 dispersed in water. Inset of (a): the left is a photograph of the corresponding GQDs-2 aqueous solution under UV light with 365 nm excitation; the right is a photograph of the corresponding GQDs-2 aqueous solution taken under visible light. (b) PL spectra of the GQDs-2 solution under different excitation wavelengths, Figure S10: PLE spectrum of GQDs-2 with the detection wavelength of 430 nm and PL spectrum excited at 328 nm, Table S1: Summary of surface element content of CTP and GQDs by XPS analysis, Table S2: Summary of C chemical bonds form in the surface of CTP and GQDs.
