# Supplementary material for: Green Preparation of High Yield Fluorescent Graphene Quantum Dots from Coal-Tar-Pitch by Mild Oxidation
Source: Nanomaterials (Basel). 2018 Oct 17;8(10):844. doi: 10.3390/nano8100844 (PMC6215232; doi:10.3390/nano8100844)
Supplement: Supplementary file 1 [file nanomaterials-08-00844-s001.pdf]

# Supplementary Information

# Green Preparation of High Yield Fluorescent Graphene Quantum Dots from Coal-Tar-Pitch by Mild Oxidation

**Quanrun Liu [1,*], Jingjie Zhang [1], He He [1], Guangxu Huang [1,2,3], Baolin Xing [1], Jianbo Jia [1] and Chuanxiang Zhang [1,*]**

[1] College of Chemistry and Chemical Engineering, Henan Polytechnic University, Jiaozuo 454003, China; zhangjingjie1221@163.com (J.Z.); hpuhehe@163.com (H.H.); guangxu1369@163.com (G.H.); baolinxing@hpu.edu.cn (B.X.); jiajianbo@hpu.edu.cn (J.J.)

[2] Collaborative Innovation Center of Coal Work Safety of Henan Province, Jiaozuo 454003, China

[3] Henan Key Laboratory of Coal Green Conversion, Jiaozuo 454003, China

***** Correspondence: qrliu@163.com (Q.L.); zcx223@hpu.edu.cn (C. Z.); Tel.: +86-10-0391-3986816 (C.Z.); Fax: +86-10-0391-3986810 (C.Z.)

**Table S1.** Summary of surface element content of CTP and GQDs by XPS analysis.

| Sample | Elements (%) | | | |
|---|---|---|---|---|
| | C | O | N | S |
| CTP | 93.54 | 3.58 | 2.62 | 0.26 |
| GQDs-1 | 69.30 | 29.84 | 0.77 | 0.09 |
| GQDs-2 | 58.85 | 40.10 | 0.98 | 0.07 |

**Table S2.** Summary of C chemical bonds form in the surface of CTP and GQDs.

| Chemical bond type | Chemical bond proportion (%) | | |
|---|---|---|---|
| | CTP | GQDs-1 | GQDs-2 |
| $Csp^2$ | 51.30 | 37.30 | 30.98 |
| $Csp^3$ | 39.49 | 23.59 | 20.35 |
| C-O | 9.21 | 12.04 | 8.00 |
| C=O | - | 5.86 | 18.28 |
| COOH | - | 21.21 | 22.37 |

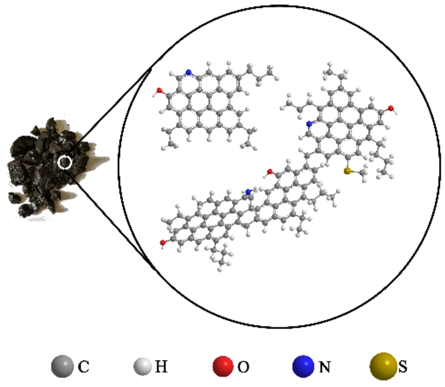


**Fig. S1.** Macroscale image and simplified illustrative molecular structure of CTP.

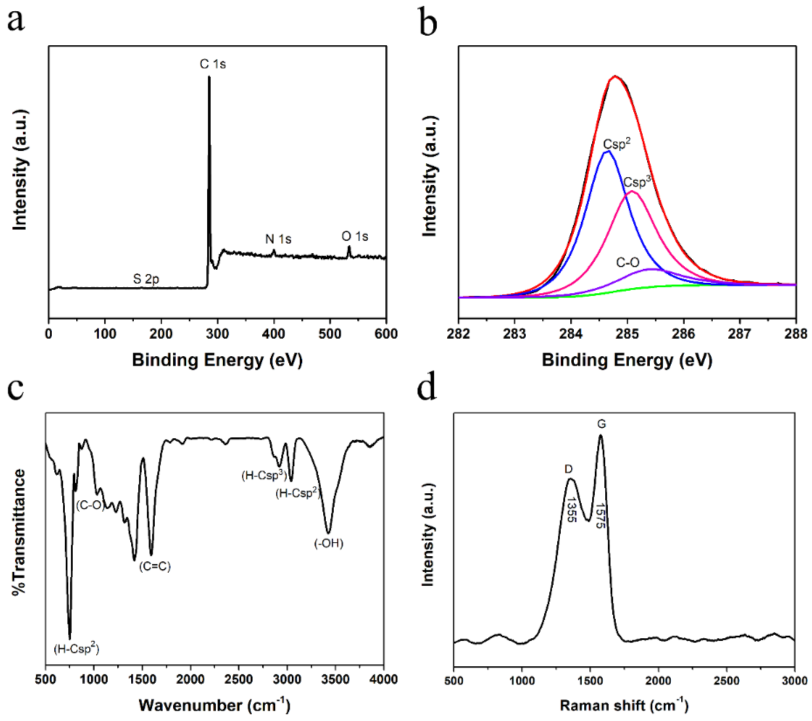

**Fig. S2.** Characterization of CTP. (a) XPS survey of CTP. (b) High resolution C 1s XPS spectrum of CTP displayed presence of $Csp^2$, $Csp^3$ and C–O modes at 284.6 eV, 285.1 eV and 285.4 eV, respectively. (c) ssFTIR spectrum of CTP showing H–$Csp^2$, C–O, C=C, H–$Csp^3$ and O–H vibration modes at 750/3040 $cm^{-1}$, 1032 $cm^{-1}$, 1593 $cm^{-1}$, 2917 $cm^{-1}$ and 3426 $cm^{-1}$ as labeled, respectively. (d) Raman spectrum of CTP under excitation of 532 nm.

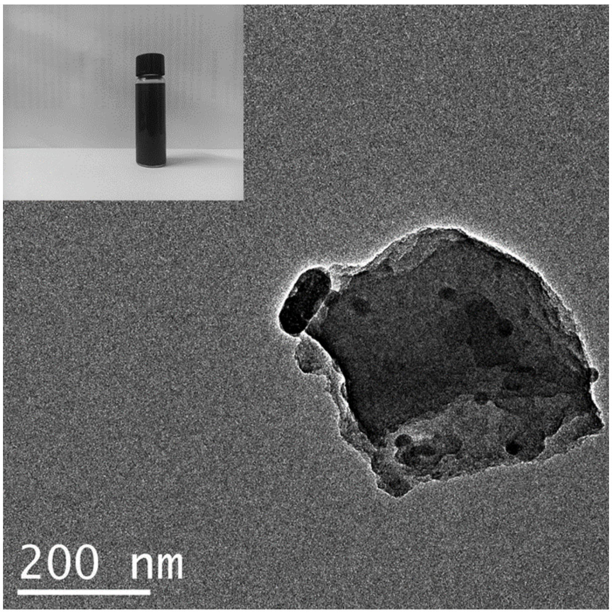


**Fig. S3.** TEM image of CTP after ultrasonication in hydrogen peroxide for 2 h. Inset is the optical image.

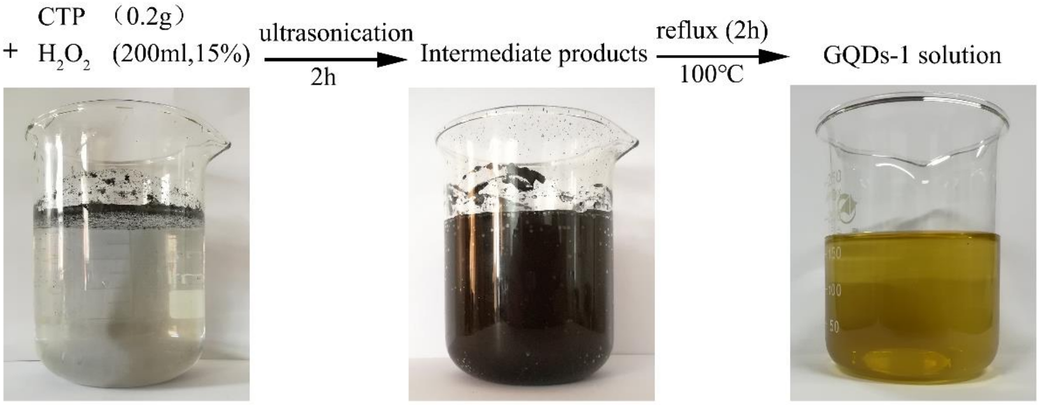


**Fig. S4.** Preparation procedure of GQDs-1.

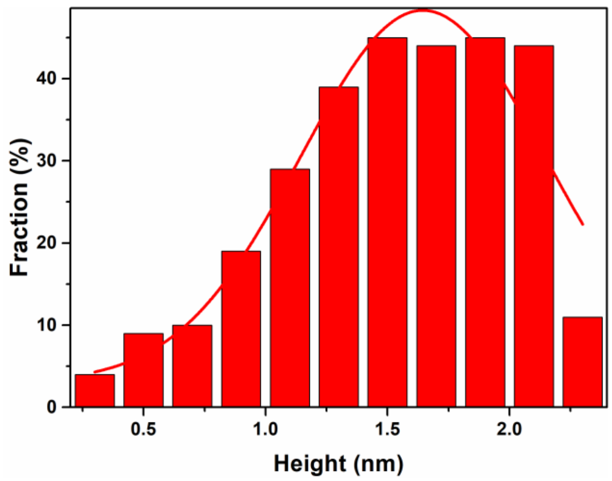


**Fig. S5.** The height distribution of the GQDs-1.

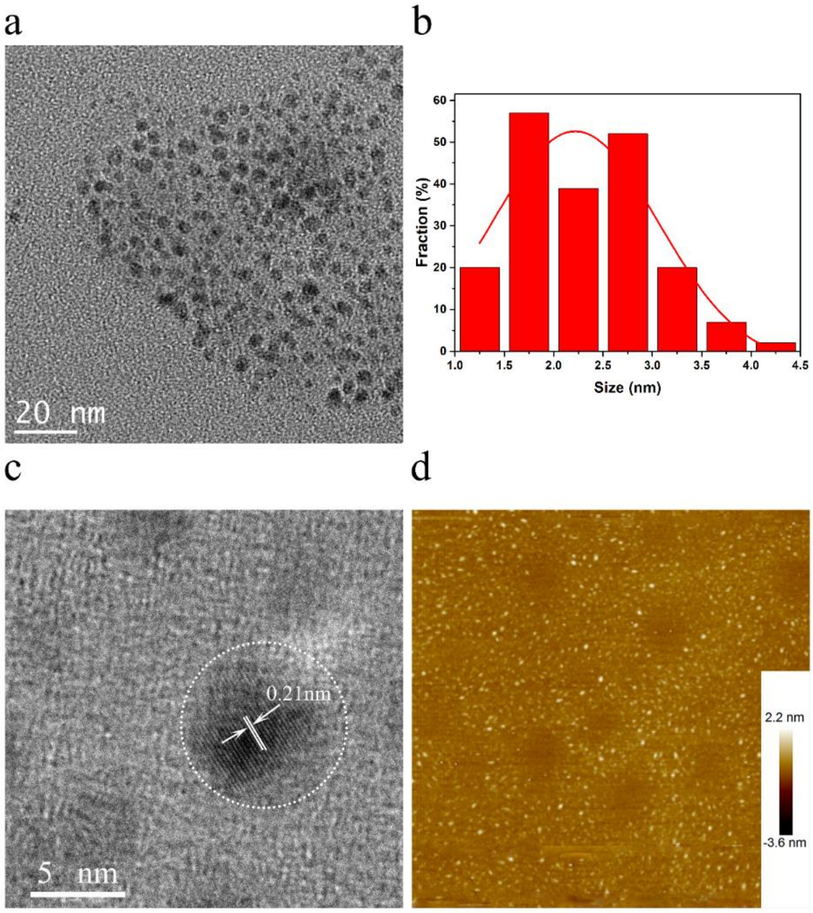


**Fig. S6.** Characterization of GQDs-2. (a) TEM image of GQDs-2 displaying a regular size and shape distribution. Scale bar, 20 nm. (b) The size distribution histogram of GQDs-2. (c) HRTEM image of representative GQDs-2 from a. (d) AFM image of GQDs-2.

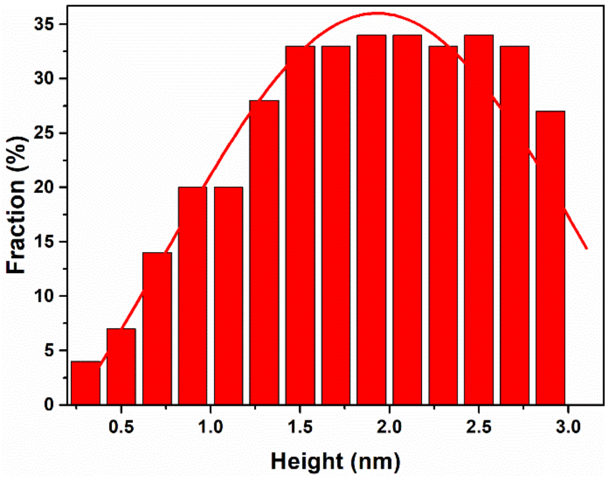


**Fig. S7.** The height distribution of the GQDs-2.

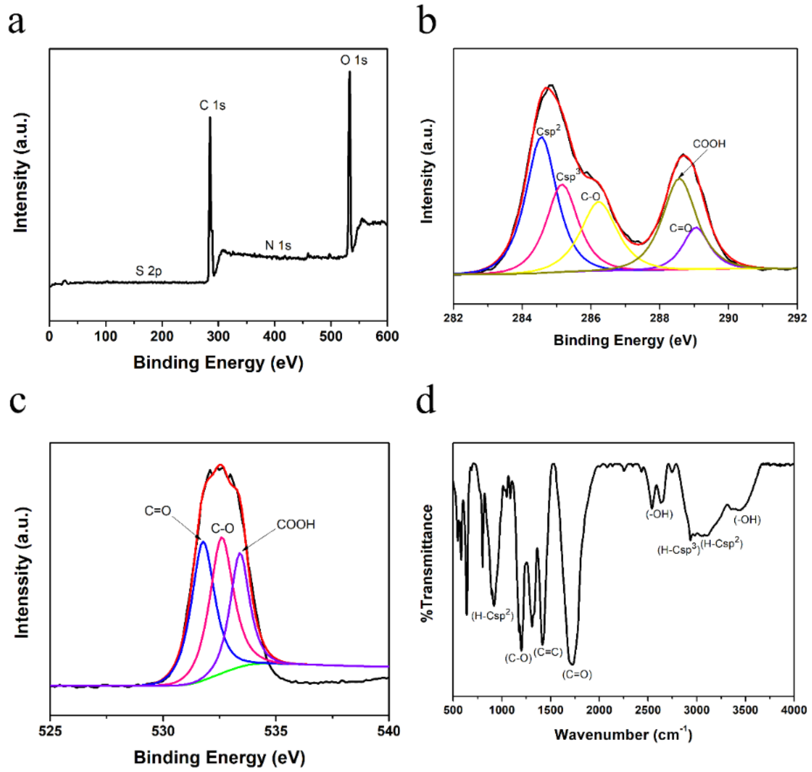


**Fig. S8.** Characterization of GQDs-2. (a) XPS survey of GQDs-2. (b) High-resolution C 1s XPS spectrum of GQDs-2. (c) The high-resolution O 1s XPS spectrum of GQDs-2. (d) ssFTIR spectrum of GQDs-2 showing different vibration modes as labeled.

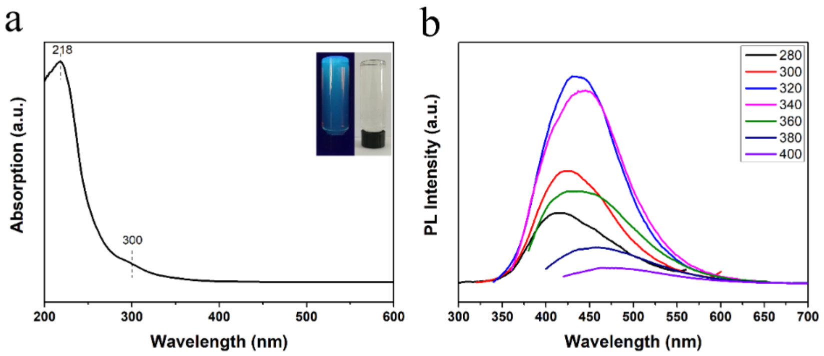

**Fig. S9.** Optical properties of the GQDs-2. (a) UV-Visible spectrum of the GQDs-2 dispersed in water. Inset of panel a: the left is a photograph of the corresponding GQDs-2 aqueous solution under UV light with 365 nm excitation; the right is a photograph of the corresponding GQDs-2 aqueous solution taken under visible light. (b) PL spectra of the GQDs-2 solution under different excitation wavelengths.

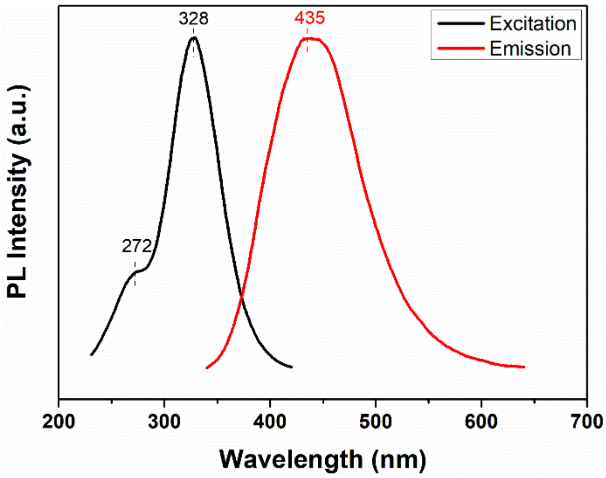


**Fig. S10.** PLE spectrum of GQDs-2 with the detection wavelength of 430 nm and PL spectrum excited at 328 nm.
